# An Imperial Pharmacopœia

**Published:** 1906-10-06

**Authors:** 


### The Hospital

A Journal of

The Medical Sciences and Hospital Administration.

Vol. XLI.—No. 1,046. SATURDAY, OCTOBER 6, 1906.

### An Imperial Pharmacopœia.

The area over which any standard fixed for the determination of the quantitative and qualitative composition of medicines shall exercise sway is a question to be decided by the strictly practical tests of convenience and utility. Into any such discussion neither sentiment nor emotion should be permitted to enter. These propositions will be readily granted by those who recognised that the purpose of a national pharmacopœia is to protect the public interest against the dangers which would inevitably arise were each individual physician and pharmacist free to define or adopt whatever standard seemed to him most appropriate. The confusion resulting from this latter method would obviously mean danger to the public, and against that danger it is the business of the Legislature, no other authority having the power, to provide protection. When, however, the Legislature has taken steps to secure this end its function in this particular direction ceases, and all further responsibility for the prescribing and dispensing of medicines must rest with the medical practitioners and pharmacists immediately concerned. From this it follows that in the last resort the influence and authority of any pharmacopœia depend upon the power and will of the governing authorities to make it operative in the region under their jurisdiction; and it may be assumed that such authorities will be guided by purely prosaic considerations and not by the pleadings of sentiment.

It seems well thus to re-state the position and function of a national pharmacopœia in view of the revival of the project for the production of a single volume which shall be enforced as the "one uniform standard and guide" in all parts of the British Empire, and to which, in somewhat flamboyant style, it is proposed to apply the title "Imperial Pharmacopœia." There can be no question that the project, and more particularly its presentation at the present date, has an alluring aspect. It appears so closely to harmonise with certain other national movements that it may be expected to attract attention and support. But from the lofty region of racial or national patriotism the proposal must descend to be tried by a utilitarian standard. If it fails here it fails altogether, and no extraneous enthusiasm can galvanise it into life.

Now it may be frankly admitted that much may be said of the convenience and advantage of the existence of one and the same standard for medicines both at home and in the Colonies. The suggestion appears to follow the course of historical development and to be merely an extension to a wider area of a principle which has already received the justification of success. The time is hardly remote when each of the three divisions of the United Kingdom possessed its own pharmacopœia, and when the London standard was authoritative in England, that of Edinburgh in Scotland, and that of Dublin in Ireland. Under such a regime a prescription received a different interpretation according as it was dispensed on one or other side of the Tweed, or to the east or west of the Irish Channel. Obviously an arrangement of this order carried risks to the medicine-taking public, and as facilities for travel and inter-communication increased these risks increased in number. Consequently, it was felt necessary to bring all parts of the country under the control and authority of a single standard, which then appeared in the shape of the British Pharmacopœia. It may now reasonably be urged that as so many persons travel to and from the Colonies the dangers of many, and in some respects conflicting, pharmacopœias are reappearing, and hence that the public safety calls out for the supersession of the numerous existing local standards by one universal imperial authority.

Whether an imperial pharmacopœia is or is not possible at the present date, no one will deny that in one respect at all events it is desirable to secure uniformity in the composition and strength of medicines. We refer to the more active and powerful members of the materia medica. And this remark applies not merely to the various countries included in the British Empire. It embraces all countries wherein a national pharmacopœia has force. When one thinks of the freedom of travel exhibited at the present day in the various countries of Europe, it is not possible to deny the risk which is attached to the different pharmacopœial standards adopted by these countries. Thus, whilst in a prescription written in England "acid. hydrocyan. dil." means a 2 per cent. solution, in France the same term would signify a solution of 1 per

199495

W1
H06 9X

cent., in Belgium 2.5, and in Spain 10 per cent. It is satisfactory to know, on the authority of Professor MacAlister, that as a result of the recent International Congress on Pharmacopœial Unification this state of matters will shortly be brought to an end, and that in all parts of the civilised world drastic or poisonous drugs and preparations will each be adjusted to a single uniform standard. When this is effected there need be little further anxiety either for a Colonial or an International Pharmacopœia. Both of these projects may come in time, but the essential claim of the situation has been met by the work of the International Congress.

